# Matrix Metalloproteinase (MMP)-9 in Cancer-Associated Fibroblasts (CAFs) Is Suppressed by Omega-3 Polyunsaturated Fatty Acids *In Vitro* and *In Vivo*

**DOI:** 10.1371/journal.pone.0089605

**Published:** 2014-02-27

**Authors:** Ayumi Taguchi, Kei Kawana, Kensuke Tomio, Aki Yamashita, Yosuke Isobe, Kazunori Nagasaka, Kaori Koga, Tomoko Inoue, Haruka Nishida, Satoko Kojima, Katsuyuki Adachi, Yoko Matsumoto, Takahide Arimoto, Osamu Wada-Hiraike, Katsutoshi Oda, Jing X. Kang, Hiroyuki Arai, Makoto Arita, Yutaka Osuga, Tomoyuki Fujii

**Affiliations:** 1 Department of Obstetrics and Gynecology, Graduate School of Medicine, The University of Tokyo, 7-3-1 Hongo, Bunkyo-ku, Tokyo, Japan; 2 Department of Health Chemistry, Graduate School of Pharmaceutical Sciences, The University of Tokyo, 7-3-1 Hongo, Bunkyo-ku, Tokyo, Japan; 3 Department of Medicine, Massachusetts General Hospital and Harvard Medical School, Charlestown, Massachusetts, United States of America; The University of Hong Kong, Hong Kong

## Abstract

Cancer associated fibroblasts (CAFs) are responsible for tumor growth, angiogenesis, invasion, and metastasis. Matrix metalloproteinase (MMP)-9 secreted from cancer stroma populated by CAFs is a prerequisite for cancer angiogenesis and metastasis. Omega-3 polyunsaturated fatty acids (omega-3 PUFA) have been reported to have anti-tumor effects on diverse types of malignancies. Fat-1 mice, which can convert omega-6 to omega-3 PUFA independent of diet, are useful to investigate the functions of endogenous omega-3 PUFA. To examine the effect of omega-3 PUFA on tumorigenesis, TC-1 cells, a murine epithelial cell line immortalized by human papillomavirus (HPV) oncogenes, were injected subcutaneously into fat-1 or wild type mice. Tumor growth and angiogenesis of the TC-1 tumor were significantly suppressed in fat-1 compared to wild type mice. cDNA microarray of the tumors derived from fat-1 and wild type mice revealed that MMP-9 is downregulated in fat-1 mice. Immunohistochemical study demonstrated immunoreactivity for MMP-9 in the tumor stromal fibroblasts was diffusely positive in wild type whereas focal in fat-1 mice. MMP-9 was expressed in primary cultured fibroblasts isolated from fat-1 and wild type mice but was not expressed in TC-1 cells. Co-culture of fibroblasts with TC-1 cells enhanced the expression and the proteinase activity of MMP-9, although the protease activity of MMP-9 in fat-1-derived fibroblasts was lower than that in wild type fibroblasts. Our data suggests that omega-3 PUFAs suppress MMP-9 induction and tumor angiogenesis. These findings may provide insight into mechanisms by which omega-3 PUFAs exert anti-tumor effects by modulating tumor microenvironment.

## Introduction

The tumor microenvironment is comprised of microvascular endothelial cells, adjacent normal epithelial cells and cancer-associated fibroblasts (CAFs), and is reported to be an important regulator of tumorigenesis [1], [2]. As the most common cellular population found in the tumor microenvironment, CAFs are responsible for the synthesis of proteins involved in the remodeling of the extracellular matrix (ECM), and for the secretion of growth factors and cytokines that regulate tumor cell proliferation and invasion [3], [4]. In murine ovarian cancer xenograft models, the p53/NF-κB pathway in CAFs significantly increased in vivo tumor growth [5]. In colon cancer, Zhu Y et al. report that IL-1β increased colon cancer cell proliferation and invasion by up-regulating COX-2 signaling in CAFs [6].

Matrix-metalloproteinases (MMPs) are synthesized as proenzymes and typically activated by proteolytic removal of a propeptide [7]. MMPs are reported to influence tumor progression by facilitating events pivotal for neovascularization and establishment of distant metastasis including proliferation, survival and migration of endothelial, tumor and stromal cells [8], [9]. MMP-2 and MMP-9 are implicated as prerequisites for angiogenesis and metastasis in the carcinogenesic process. MMP-2 is expressed in the various cancer cell lines [10]. In contrast, MMP-9 has very limited or no expression in these cancer cells. Instead, MMP-9 is well-known to be secreted from cancer stromal fibroblasts and endothelial cells [11], [12]. MMP-9 is a member of a family of zinc containing endoproteinases that is involved in degradation of extracellular matrix (ECM) and in vascular remodeling [13].

Docosahexaenoic acid (DHA, 22∶6n-3) and eicosapentaenoic acid (EPA, 20∶5n-3) are representative mediators of omega-3 polyunsaturated fatty acids (omega-3 PUFAs) and exert anti-inflammatory effects in acute and chronic pathological inflammatory reactions by counteracting inflammation [14]. Omega-3 PUFAs are also reported to have anti-cancer effects based on in vitro and vivo studies [15]–[17]. Several mechanisms have been proposed to explain the anti-cancer effects of omega-3 PUFAs. Omega-3 PUFAs alter the growth of tumor cells by modulating cell replication, by interfering with components of the cell cycle or by increasing cell death via necrosis or apoptosis [18], [19]. Omega-3 PUFAs are also known to exert anti-angiogenic effects by inhibiting the production of many angiogenic mediators including: vascular endothelial growth factor (VEGF), platelet-derived growth factor (PDGF), and prostaglandin E2 (PGE2) [20]–[24].

Dietary supplementation is a traditional approach to modify tissue nutrient composition in animal studies of nutrition. Feeding animals diets that alter specific nutritional and non-nutritional components can help to differentiate experimental groups; however, it can be exceedingly difficult to provide diets that are identical in all but a single or a small but controlled number of components. Kang et al. recently engineered a transgenic mouse that carries the fat-1 gene from the roundworm *Caenorhabditis elegans* [25]. This gene encodes an omega-3 fatty acid desaturase that catalyzes the conversion of omega-6 to omega-3 PUFAs and that is absent in most animals, including mammals. There is a remarkable difference in the tissue omega-6/omega-3 PUFA ratio between wild type and fat-1 transgenic mice [26]. Fat-1 mice, which typically exhibit a balanced ratio of omega-6 to omega-3 PUFAs in their tissues and organs independent of diet, allow carefully controlled studies to be performed in the absence of potential confounding factors of diet. This makes them a useful model to investigate the biological properties of endogenous omega-3 PUFAs [25]. Several reports using fat-1 mice have demonstrated anti-cancer effects of omega-3 PUFAs [27]–[30]. In these investigations, omega-3 PUFAs exerted anti-cancer effects by suppressing inflammatory reactions and PGE2 secretion from cancer cells. To date, there are few studies that investigate the involvement of omega-3 PUFAs in the biology of CAFs.

In this study, we hypothesized that omega-3 PUFAs may alter tumor microenvironments by influencing CAF activity. To examine this hypothesis, fibroblasts derived from fat-1 and wild type mice were assessed both in vitro and in vivo under conditions allowing interaction with TC-1 cancer cells. TC-1 cells were derived from the epithelium of C56BL/6 mice and immortalized by human papillomavirus (HPV) type 16 E6 and E7 oncoproteins. They are commonly used in vitro and in vivo in murine models of HPV-related cancer [31]. Here, we investigated the involvement of omega-3 PUFAs in TC-1 tumorigenesis by comparing fat-1 and wild type mice. Our specific focus involved the study of tumor-associated fibroblasts. These models are useful in the study of CAFs because the cancer cells originate in wild type murine epithelium while the cancer stromal components, including CAFs, come from fat-1 (omega-3 PUFAs-rich) or wild type (normal PUFAs) mice.

## Materials and Methods

### Animals and diet

Fat-1 mice were created on a C57BL/6 background as described [26] and subsequently backcrossed (at least four times) onto a C57BL/6 background. Animals were fed a special diet (AIN-76A+10% safflower oil; CLEA Japan, Inc.) that contained 10.3% total fat with fatty acid composition of C16∶0 (7.6%), C18∶0 (2.7%), C18∶1n-9 (14.1%), C18∶2n-6 (73.2%), C18: 3n-3 (0.3%), C20∶4n-6 (<0.1%), C20∶5n-3 (<0.1%), C22: 6n-3 (<0.1%), high in n-6 and low in n-3 fatty acids, until the desired age (6–8 weeks) for experiments. To prevent the oxidation of lipids in the diet, all foods were stored in the refrigerator with antioxidants (AGELESS; Mitsubishi Gas Chemical Inc.), and prepared newly every two days. Animal studies were approved by the University of Tokyo Animal Committee.

### Tumor growth assay in mice

TC-1 cells are derived from a primary lung epithelial cell from C56BL6/mice and immortalized using HPV 16 E6/E7 plus c-Has-ras (kind gift from Dr. T. C. Wu, Johns-Hopkins University, Baltimore MD USA) [32]. TC-1 cells were cultured in DMEM (Gibco, NY, USA) containing 10% FBS, 100 U/ml penicillin, 0.1 mg/ml streptomycin, and 0.25 g/ml amphotericin B. Eight-week-old female mice were injected with 5×10^6^ murine TC-1 cells suspended in 100 µl of DMEM. Tumor volume, based on caliper measurements, was calculated at 7 and 14 days after injection according to the following formula: (tumor volume) = 1/2×(the shortest diameter) ^2^×(the largest diameter). Mice were sacrificed 14 days after inoculation, tumors were excised and stored at −80°C for future analyses.

### cDNA microarray

Total RNA from TC-1 tumors (above) was extracted using an RNeasy minikit (QIAGEN, Hilden, Germany). For the cDNA microarray analysis, 0.5 µg of pooled total RNA was amplified and labeled using an Amino Allyl MessageAmpTM II mRNA Amplification kit (Applied Biosystems, Foster City, CA, USA) according to the manufacturer's instructions. Each sample of mRNA labeled with Cy3 and reference mRNA labeled with Cy5 was cohybridized to the GeneTM Mouse Oligo chip 24 k (Toray Industries Inc., Tokyo, Japan) at 37°C for 16 h. After hybridization, each DNA chip was washed and dried. Hybridization signals derived from Cy3 and Cy5 were scanned using Scan Array Express (PerkinElmer, Waltham, MA, USA). The scanned image was analyzed using GenePix Pro (MDS Analytical Technologies, Sunnyvale, CA, USA). All analyzed data were scaled by global normalization. GEO accession number is GSE54079.

### Immunohistochemistry

Paraffin sections (4 µm) of TC-1 tumors were dewaxed in xylene and rehydrated through graded ethanol to water. Antigens were retrieved by boiling in 10 mM citrate buffer (pH 6.0) for 30 min. The cooled sections were incubated in DAKO REAL Peroxidase-Blocking solution (DAKO, Carpinteria, CA, USA) for 10 min to quench endogenous peroxidase. To block nonspecific binding, sections were incubated in DAKO Protein Blocking solution (DAKO) for 10 min at room temperature. Sections were then incubated with a rabbit polyclonal antibody against mouse MMP-9 (PAB12714, Abnova, 1∶100 dilution) in DAKO REAL Antibody Diluent (DAKO) overnight at 4°C. The slides were incubated for 1 hour at room temperature with peroxidase-conjugated secondary antibodies, washed, incubated with DAB, counterstained with hematoxylin, dehydrated through an ethanol series and xylene, and mounted. To evaluate tumor microvessel formation, tumor sections were stained for CD-31 using a rat monoclonal antibody against mouse CD-31 (ab56299, Abcam, Tokyo, Japan, 1∶100 dilution).

### RT-quantitative PCR (RT-qPCR)

Total RNA was extracted from TC-1 tumors and cultured fibroblasts using an RNeasy minikit (QIAGEN, Hilden, Germany), followed by reverse transcription. cDNA was amplified for 40 cycles in a Light Cycler 480 (Roche, Basel, Switzerland) using a Universal Probe Master Mix and the following primers and Universal Probe Library (UPL) probes (Roche). The primer pairs and the universal probes corresponding to the each primer that were used in amplifications were as follows: mouse β-actin, 5′- ATTGAAACATCAGCCAAGACC-3′ and 5′-CCGAATCTCACGGACTAGTGT-3′ probe88; mouse MMP-9, 5′-ACGACATAGACGGCATCCA-3′ and 5′-GCTGTGGTTCAGTTGTGGTG-3′ probe19. Expression of MMP-9 was normalized using β-actin mRNA as an internal standard. Expression levels were calculated by the comparative Ct method using β-actin as an endogenous reference gene.

### Primary fibroblast culture and co-culture with TC-1 cells

Lungs were isolated from fat-1 transgenic and wild type mice and washed with saline to remove blood cells. Isolation and culture of pulmonary fibroblasts were performed using methods described previously [33]. Lung tissues were minced into small pieces and incubated in DMEM (Gibco, NY, USA) containing type I collagenase (0.25%; Sigma-Aldrich, St Louis, MO, USA) and deoxynuclease I (15 U/ml; TaKaRa, Tokyo, Japan) for 120 min at 37°C. The resultant dispersed cells were separated by filtration through nylon cell strainers (70 µm, BD, Franklin Lakes, NJ, USA). Fibroblasts in the filtrate were collected, placed into 10 cm dishes in DMEM containing 10% FBS, 100 U/ml penicillin, 0.1 mg/ml streptomycin, and 0.25 mg/l amphotericin B, and incubated for 7–10 days. Fibroblasts were purified from other cell population by differential adhesion and serial passage.

Confluent fibroblasts and TC-1 cells were trypsinized and resuspended in DMEM containing 10% FBS, 100 U/ml penicillin, 0.1 mg/ml streptomycin, and 0.25 g/ml amphotericin B and 1×10^5^ cells/ml cells of each cell type were plated together in 12 well culture plates. Co-cultures were incubated at 37°C 5% CO2 in a humidified atmosphere for 24 hours. Homotypic cultures served as controls.

### Gelatin zymography

Gelatin zymography assays were performed using a Gelatin zymography kit (Cosmobio, Sapporo, Japan) according to the manufacture's instuctions. Cell culture supernatants were collected and centrifuged at 1,500 rpm for 5 minutes. The cell free supernatant was mixed with 2× sample buffer and electrophoresed using precast gels (10% polyacrylamide, 0.1% gelatin) at 4°C for 1 hour. Subsequentnzymatic reactions were performed at 37°C overnight. Gelatinase activities were visualized using specific staining solutions and destained in acetic acid-methanol-dH_2_O (1∶3∶6). For semi-quantitative analyses, gelatin zymography bands were analyzed using image analysis software (ImageJ).

### Statistical analysis

Data are presented as means ± SEM. Statistical analyses were carried out by Student's *t*-test, or Wilcoxon analysis using JMP software. A value of P<0.05 was considered significant. In the figure legends, asterisks indicate those comparisons with statistical significance (p<0.05).

## Results

### Tumor growth and angiogenesis of TC-1 tumors is suppressed in fat-1 mice

**Figure 1:**
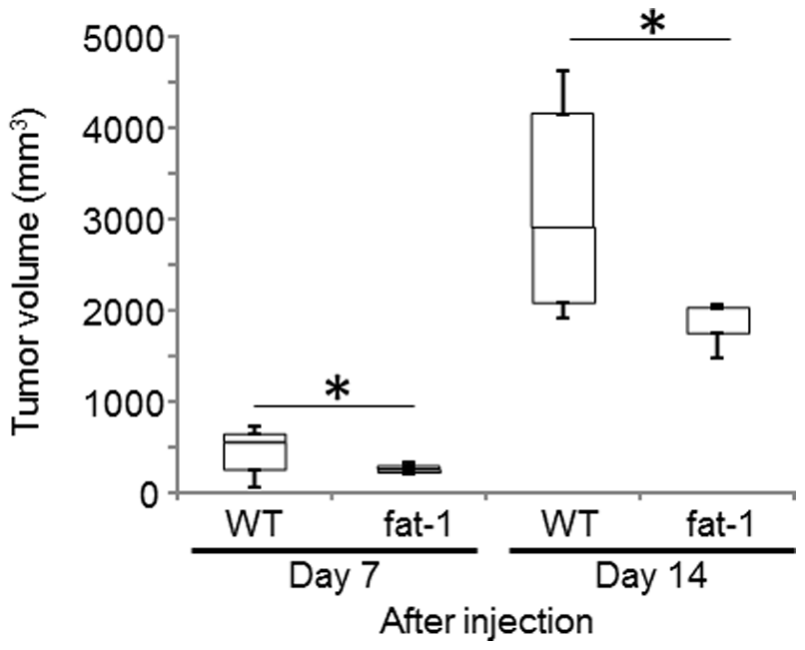
Tumor growth rates in fat-1 and wild type (WT) mice. 5×10^6^ murine TC-1 cells suspended in 100 µl of DMEM were injected s.c. into each of 10 fat-1 and wild type mice. Tumor volume, based on caliper measurements, was calculated at 7 and 14 days after injection according to the following formula: (tumor volume) = 1/2×(the shortest diameter) ^2^×(the largest diameter). Mean values with standard deviations are presented. Asterisks indicate those comparisons (fat-1 vs. wild type mice) with statistical significance (p<0.05).

To investigate the effect of omega-3 PUFAs on cervical cancer tumorigenesis, we injected TC-1 cells subcutaneously into fat-1 and the litter-mate wild type C57/BL6 mice. TC-1 tumor formation rates and tumor growth were assessed by the number of mice forming tumors and three-dimension tumor sizes, respectively. There was no difference in tumor formation rates between fat-1 and wild type mice. TC-1 tumor sizes were plotted for 14 days in fat-1 and wild-type controls (Fig. 1). Tumor growth rates were consistently lower in fat-1 mice when compared to wild type mice at all time points. In fat-1 mice, tumor size at 14 days after injection was significantly smaller than in wild-type controls (Fig. 1). Although cell growth of TC-1 cells is dependent on HPV16 E6/E7 expression, E6 and E7 expression in TC-1 tumors was not downregulated in fat-1 mice (Fig. S1).

**Figure 2:**
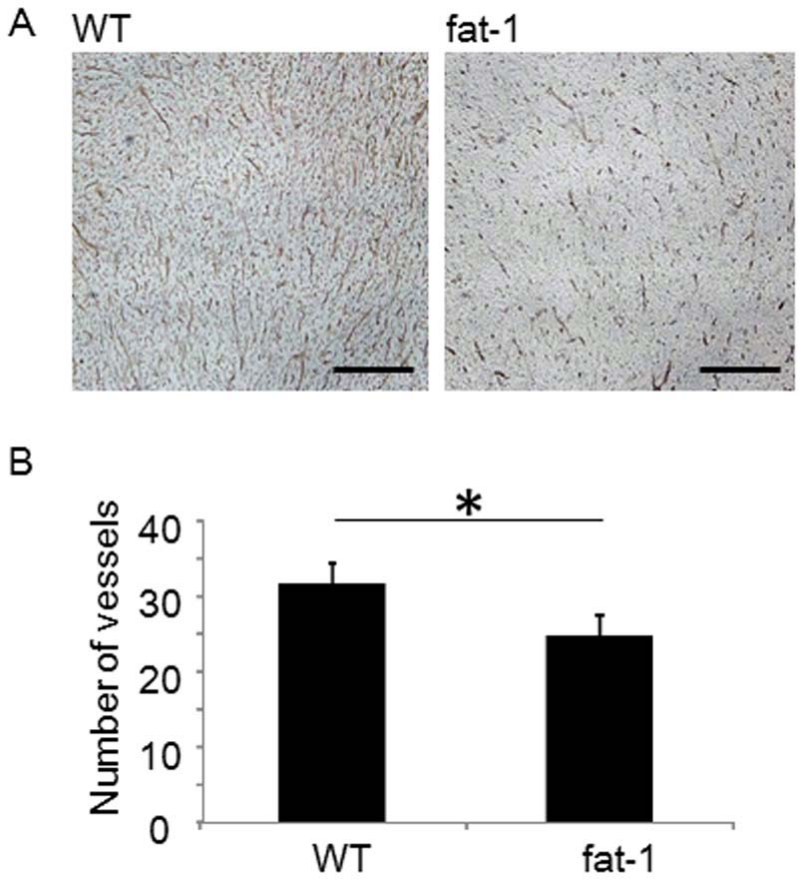
Omega-3 PUFAs suppress tumor vasculogenesis. CD31 immunostaining of the TC-1 tumor derived from wild type (WT) mice (A) and fat-1 (B). Bars indicate 200 µm. (C) Microvessel densities in TC-1 tumors are expressed as the representative number of labeled vessels in 4 fields (n = 5). Mean values with standard deviations are presented. Asterisks indicate those comparisons (fat-1 vs. wild type mice) with statistical significance (p<0.05).

To further delineate mechanisms behind these differences in tumor growth in this model, particularly in terms of the possible role of host-derived cancer-associated stromal components including CAFs, we next examined whether omega-3 PUFAs modified angiogenesis in TC-1 tumor. We assessed semi-quantitatively tumor microvessel density in TC-1 tumors derived from fat-1 and wild type mice by counting the number of CD31-positive microvessels in immunohistochemical assay (Fig. 2A and 2B). CD31 immunostaining of TC-1 tumors derived from fat-1 and wild type mice (Fig. 2A) demonstrated hypovascularity of the fat-1-derived TC-1 tumors when compared with wild type-derived tumors. The number of CD31-positive microvessels per high-power field in fat-1 mice was significantly lower than that in wild type mice (Fig. 2B). These in vivo data indicated that TC-1 tumor growth and angiogenesis were at least suppressed in fat-1 mice when compared with wild type counterparts although it was difficult to accurately estimate TC-1 cell growth in fat-1 mice.

### MMP-9 is downregulated in fat-1 mice-derived TC-1 tumors

**Figure 3:**
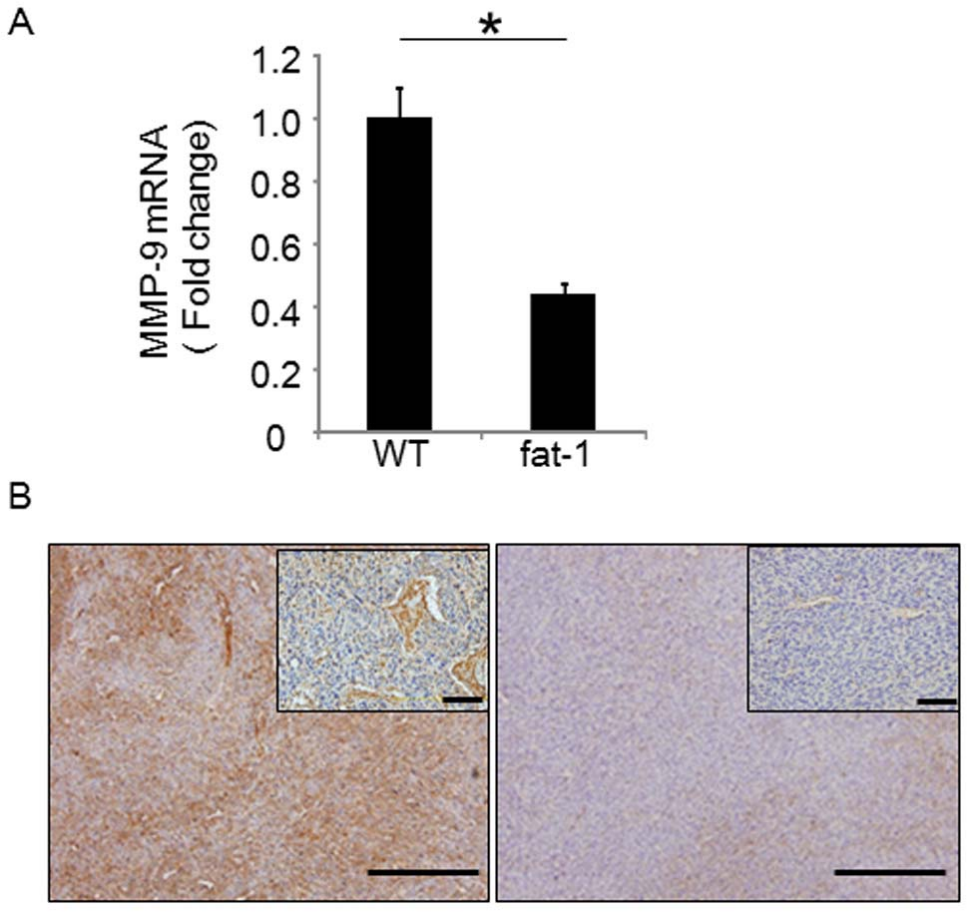
MMP-9 expression is downregulated in TC-1 tumors from fat-1 mice. Total RNA was extracted from TC-1 tumors, followed by reverse transcription. MMP-9 mRNA levels were measured by qRT-PCR. Expression levels of MMP-9 were normalized to β-actin as an internal standard (n = 4 in each group). Asterisks indicate those comparisons (fat-1 vs. wild type (WT) mice) with statistical significance (p<0.05). MMP-9 immunostaining of TC-1 tumors derived from wild type (WT) and fat-1 mice. Bars indicate 200 µm in low-power fields, 50 µm in high-power fields.

To investigate potential differences in gene expression profiles of TC-1 tumors growing in the skin of fat-1 and wild type mice, TC-1 tumor tissues obtained from fat-1 and wild type mice were analyzed by cDNA microarray. Since omega-3 PUFAs are reported to influence cell proliferation and inflammation, Table 1 lists relative gene expression levels for representative genes associated with the tumor growth and inflammation (Table 1). With the exception of EGF, expression levels of almost all inflammatory cytokines/chemokines and growth factors in TC-1 tumors from fat-1 mice were higher than those, in tumors from wild-type controls. This suggested that anti-inflammatory and anti-cell growth effects of omega-3 PUFAs are unlikely to be central to their anti-tumor activities, at least in this model. We next examined the expression of MMPs in these TC-1 tumors to further address stroma-related angiogenesis in the tumor microenvironments (Table 1). cDNA microarray demonstrated that the expression of MMP-2 and -9 were suppressed in fat-1 mice-derived TC-1 tumor while those of other MMPs were tended to be upregulated when compared to controls. To confirm these effects at the RNA level, RT-qPCR for MMP-9 was performed. MMP-9 RNA levels in fat-1 mice were approximately 60% lower than those in wild type controls (Fig. 3A). TC-1 tumor immunohistochemistry confirmed these results at the protein level. MMP-9 immunoreactivity in wild type mouse-derived TC-1 tumors was clearly stronger than fat-1 mouse-derived tumors (Fig. 3B). High power histochemical analysis of MMP-9 immnoreactivity in TC-1 cells (Fig. 3B, inserts) revealed negligible expression, while that of the stromal components including CAFs and endothelial cells was strongly positive only in the wild type-derived TC-1 tumors. These data indicated that the production of MMP-9 in CAFs and endothelial cells was clearly suppressed in fat-1 mice.

**Table 1:** Cytokine, growth factor, and MMP gene expression comparisons in TC-1 tumors from fat-1 vs wild type mice.

Genes	fat-1/WT ratio
**EGF**	**0.4**
CXCL-12	1.2
HGF	1.3
TGF-α	1.4
IL-6	2.0
IFN-γ	2.4
TNF-α	3.3
VEGF	3.7
IL-1β	12.0
**MMP-9**	**0.4**
**MMP-2**	**0.6**
MMP-7	1.9
MMP-1b	2.9
MMP-3	2.9
MMP-13	2.9
MMP-1a	3.6
MMP-16	4.1
MMP-10	11.6

### MMP-9 expression and gelatinase activity were suppressed in cultured primary fibroblasts derived from fat-1 mice

**Figure 4:**
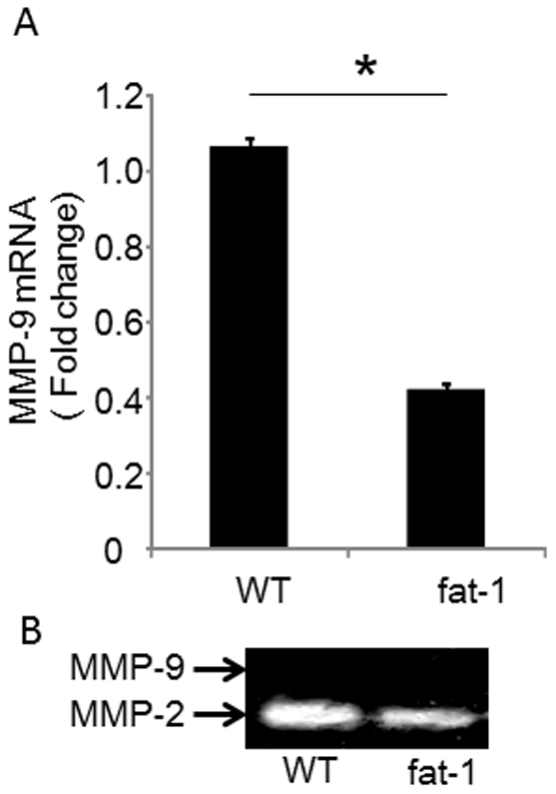
MMP-9 expression and enzymatic activity in primary-cultured fibroblasts. (A) Primary fibroblasts isolated from murine lungs were cultured. Total RNA from fibroblasts was reverse transcribed and MMP-9 mRNA levels were measured by qRT-PCR. Expression levels of MMP-9 were normalized to β-actin as an internal standard. Data are the representative of three independent experiments. The data were analyzed by using the Student's *t*-test. Asterisks indicate those comparisons (fat-1 vs. wild type (WT) mice) with statistical significance (p<0.05). (B) Gelatin zymography: Supernatant from primary fibroblast cultures were collected and separated by electrophoresis. Gelatinase activities were visualized by standard staining techniques.

To mimic the cancer stromal microenvironment in vitro, we co-cultured fibroblasts isolated from fat-1 and wild type mice with TC-1 cells. We used lung tissues from fat-1 and wild type mice as the sources for fibroblasts, and differential adhesion methodology for their isolation. All fibroblasts were passaged 3–4 times prior to use in experiments. Baseline MMP-9 expression levels in primary fibroblasts from fat-1 mice were approximately 60% lower than those from wild type-derived fibroblasts (Fig. 4A). Culture supernatants from each primary fibroblast subtype were collected and subjected to polyacrylamide gel electrophoresis to examine differences in MMP gelatinase activities (Fig. 4B). Using this method, MMP-2 was detected but MMP-9 was not in both fat-1 and wild type fibroblast

**Figure 5:**
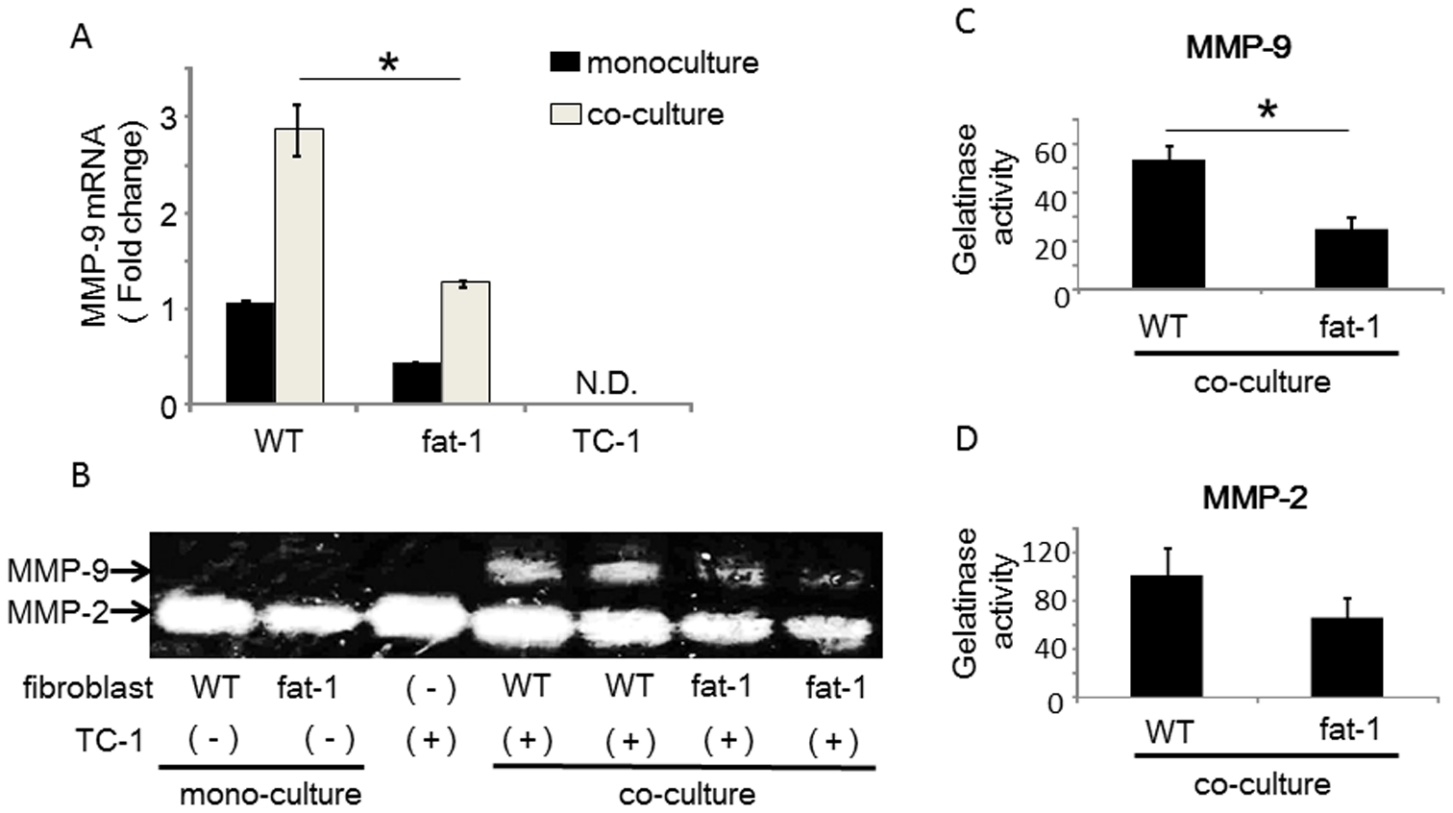
The increased MMP-9 expression and activity in TC-1/fibroblast co-cultures is inhibited in fat-1 mice. (**A**) Isolated fibroblasts were co-cultured with TC-1 cells for 24 hours and expression levels of MMP-9 in the fibroblasts were measured by RT-qPCR. Expression levels of MMP-9 were normalized to β-actin as an internal standard. The data are representative of three independent experiments. The data were analyzed using the Student's *t*-test. Asterisks indicate those comparisons (fat-1 vs. wild type (WT) mice) with statistical significance (p<0.05). “N.D.” indicates ‘not detected’. (B) Gelatin zymography: Supernatants from fibroblast homotypic cultures and fibroblast/TC-1 co-cultures were collected and separated by electrophoresis. Gelatinase activities were visualized by standard staining techniques. (C, D) For semi-quantitative analyses, gelatin zymography bands were analyzed using image analysis software. Results are represented as mean ±SEM of three independent experiments. The data were analyzed using the Student's *t*-test. Asterisks indicate those comparisons (fat-1 vs. wild type (WT) mice) with statistical significance (p<0.05).

Next, these primary fibroblasts were co-cultured with TC-1 cells to examine fibroblasts activation upon in vitro exposure to cancer cells ^11^. Fibroblasts and TC-1 cells were co-cultured for 24 hours and MMP-9 transcription was measured by RT-qPCR. Fibroblasts from fat-1 and from wild type mice increased demonstrated MMP-9 expression upon exposure to TC-1 cells (Fig. 5A). In fibroblasts derived from fat-1 mice, the extent of MMP-9 induction was lower than that in fibroblasts from wild type mice (Fig.5A). MMP-9 was not expressed in homotypic TC-1 cells, consistent with immunohistochemical data from our in vivo model. Furthermore, we confirmed that MMP-9 was not expressed in the TC-1 cells by using a transwell co-culture model (Fig. S2). Therefore, MMP-9 in the co-culture condition was derived not from TC-1 cells but from fibroblasts. MMP-2 gelatinase activity was detected in all cell culture conditions, including homotypic TC-1 cell cultures, and was not altered by co-culture conditions (Fig 5B, 5D). MMP-9 gelatinase activities were, however, detectable in fibroblast-TC-1 cell co-cultures, although MMP-9 gelatinase activity involving fat-1 fibroblasts was clearly suppressed when compared with wild type fibroblasts (Fig 5B, 5C), again supporting the concept that MMP-9 expression and gelatinase activity are suppressed by endogenous omega-3 PUFAs in CAFs derived from fat-1 mice.

## Discussion

In this study, we examined the involvement of defined dietary factors (omega-3 and omega-6 PUFAs) in tumorigenesis using HPV-positive TC-1 cells and proposed a novel anti-tumor mechanism for omega-3 PUFAs that depends on the activities of CAFs. Omega-3 PUFAs have been shown to suppress cancer incidence and growth in various types of cancers [18], [19], [27]. Many studies have demonstrated that omega-3 PUFAs have these effects through anti-inflammatory responses directly and/or indirectly [25]–[27] as well as through inducing tumor cell apoptosis and/or suppressing tumor cell proliferation [18], [19]. However, there have been few reports about the effects of omega-3 PUFAs on CAFs. CAFs have been implicated in facilitating the growth of several tumors by directly stimulating tumor cell proliferation and by enhancing angiogenesis [34], [35]. Targeting genes and signaling pathways mediating interaction of CAFs and tumor-microenvironment are considered to be essential for development of new and effective cancer therapies [36], [37]. In this study, using fat-1 mice and TC-1 tumor cells, we were able to clarify the effect of omega-3 PUFA-rich CAFs on tumorigenesis.

Many molecules, including growth factors, cytokines, and MMPs, play stimulatory and inhibitory roles in promoting angiogenesis [21]. We investigated gene expression of angiogenesis-related cytokines, growth factors and MMPs in the TC-1 tumors of fat-1 and wild type mice by cDNA microarray. The expression levels of almost all inflammatory cytokines/chemokines and growth factors in TC-1 tumors from fat-1 mice were higher than those in tumors from wild-type controls. In contrast, EGF and MMP-2 and -9 expression levels in fat-1 mice were lower than those in wild type mice. Since down-regulation of EGF in TC-1 tumors from fat-1 mice was predicted to promote TC-1 cell proliferation but tumor size in fat-1 mice was significantly smaller than in wild-type controls, we hypothesize that differences in MMP production between fat-1 and wild type mice may be responsible for the suppression of TC-1 tumor growth. Our in vitro data verified that MMP-9 derived from CAFs activated by TC-1 cell exposure was downregulated in the fat-1 mice. MMPs are reported to influence tumor progression by facilitating events pivotal for neovascularization and for the establishment of distant metastasis including proliferation, survival and migration of endothelial, tumor and stromal cells [8], [9]. MMP inhibitors reduce angiogenesis, tumor number, and tumor growth, as does genetic ablation of MMP-9 [38]. In contrast to MMP-2, which is constitutively expressed, MMP-9 levels are usually low and enzyme expression is induced by cytokines that stimulate NF-κB [8], [39]. Furthermore, elevated serum and tissue levels of MMP-9 are reported to be associated with cancer invasion and metastasis [40]. In breast cancer, MMP-9 activity has been localized around CAFs and CAFs co-cultured with cancer cells which secrete TGF-β, TNF-α, and other cytokines, increase production of MMP-9 [11], consistent with our in vitro data. In our data, suppression of MMP-9 would contribute to accompany hypo-angiogenesis in the tumor stromal component in fat-1 tumor.

Several transcription factors, including activator protein-1 (AP-1), Sp-1, and NF-κB, are reported to be involved in alterations in MMP-9 expression following exposure to various cytokines [41]–[43]. Conversely, several reports have demonstrated that omega-3 PUFAs have an inhibitory effect on the NF-κB pathway when activated by various stimuli [43]–[47]. Our own data suggest that activation of the NF-κB pathway upon co-culture with TC-1 cells may be suppressed by elevated level of omega-3 PUFAs in fibroblast obtained from fat-1 mice.

In our experiments, we consistently used primary fibroblasts that had been passaged 3-4 times only. Nevertheless, lipid mediator analysis of the fibroblasts revealed that those derived from fat-1 mice produced larger amounts of omega-3 PUFAs including EPA-derived metabolites when compared with those derived from wild type controls (Fig.S3), confirming that the cultured fibroblasts retained the characteristic to make omega-3 PUFA rich environment.

TC-1 tumor microarray data showed that several inflammatory cytokines/chemokines and growth factors were upregulated in fat-1 mice. However, the data indicated gene expression levels in both TC-1 cells and stromal components, including fibroblasts, endothelial and immune cells. Therefore, expression levels of each cytokine/chemokine were dependent on their primary sources of production. MMP-9 was produced by the fibroblasts derived from fat-1 mice but not by TC-1 cells. Therefore, suppression of the NF-κB pathway by elevated levels of omega-3 PUFAs in fat-1-derived stromal components may have a specific and potent effect on MMP-9 expression levels when compared with the other inflammatory cytokines/chemokines. On the other hand, a previous study demonstrates that omega-3 PUFAs activate NK cells and increase proportions of activated CD8+ cells; this is followed by enhanced anti-tumor effects [48]. Therefore, omega-3 PUFAs may exert both anti-inflammatory and pro-inflammatory effects on immune cells.

In this study, we have demonstrated that an omega-3 PUFAs-rich microenvironment can suppress MMP-9 secretion from CAFs and that this is associated with subsequent tumor hypo-angiogenesis. This study proposes a novel anti-tumor effect of omega-3 PUFAs by modulating tumor microenvironment especially on CAFs.

## Supporting Information

Figure S1 **Expression levels of E6 and E7 mRNA in TC-1 tumor.** Total RNA was extracted from TC-1 tumors, followed by reverse transcription. E6 and E7 mRNA levels were measured by qRT-PCR. Expression levels of E6 and E7 mRNA were normalized to β-actin as an internal standard. The E6 primers were forward, 5'- TGCACAGAGCTGCAAACAAC -3', and reverse, 5'- AGCATATGGATTCCCATCTC -3'. The E7 primers were forward, 5'- TTTGCAACCAGAGACAACTGA -3', and reverse, 5'- GCCCATTAACAGGTCTTCCA -3'.
(TIF)

Figure S2 **MMP-9 mRNA was not induced from TC-1 cells by co-culture with fibroblasts.** 150 µL of suspensions (2×10^6^/mL) of TC-1 cells or fibroblasts were added to the upper chamber, 500 µL of suspensions (1×10^5^/mL) of TC-1 cells or fibroblasts were added to the lower chamber of the 24 well Transwell plates (1 µm pore) and placed in an incubator with 5% CO_2_ at 37°C for 24 h and expression levels of MMP-9 in the TC-1 or fibroblasts in the lower chamber were measured by RT-qPCR. N.D. indicates ‘not detected’.
(TIF)

Figure S3 **Lipid mediator analysis of fibroblast/TC-1 co-cultured medium.** Supernatants from fibroblast/TC-1 co-cultures were collected and LC-MS/MS-based mediator lipidomics was performed on Acquity UPLC BEH C_18_ column (1.0 mm×150 mm×1.7 µm) using Acquity UltraPerformance LC system (Waters Co.) coupled to an electrospray (ESI) triple quadrupole mass spectrometer (QTRAP5500; AB SCIEX). The MS/MS analyses were performed in negative ion mode, and the eicosanoids and docosanoids were identified and quantified by multiple reaction monitoring. Calibration curves between 1 and 1000 pg and the LC retention times for each compounds were constructed with synthetic standards.
(TIF)
