# Supplementary figures and images for: Matrix Metalloproteinase (MMP)-9 in Cancer-Associated Fibroblasts (CAFs) Is Suppressed by Omega-3 Polyunsaturated Fatty Acids In Vitro and In Vivo
Source: PLoS One. 2014 Feb 27;9(2):e89605. doi: 10.1371/journal.pone.0089605 (PMC3937340; doi:10.1371/journal.pone.0089605)

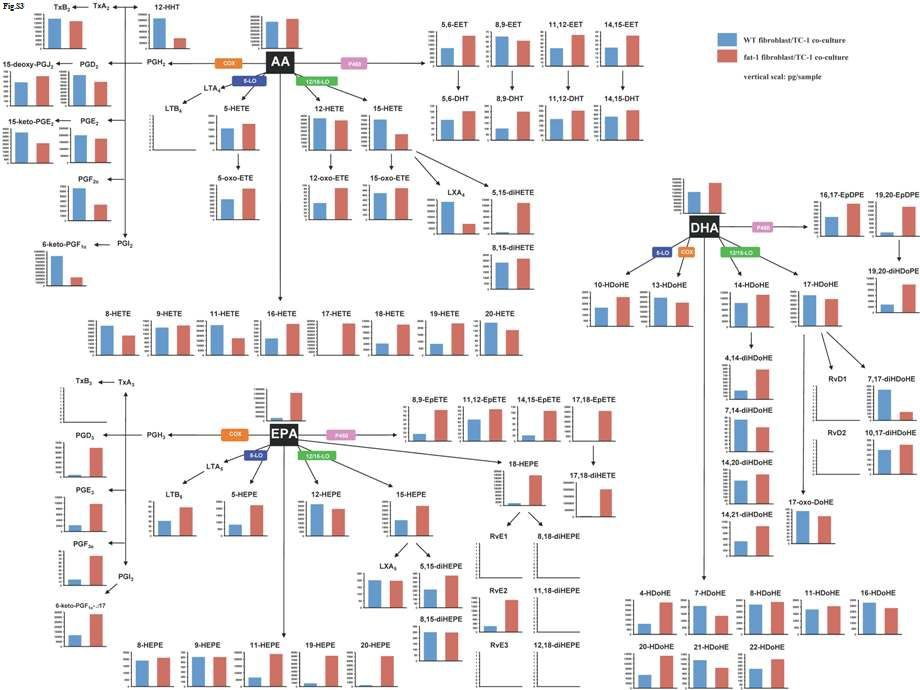

Supplement: Figure S3 — Lipid mediator analysis of fibroblast/TC-1 co-cultured medium. Supernatants from fibroblast/TC-1 co-cultures were collected and LC-MS/MS-based mediator lipidomics was performed on Acquity UPLC BEH C18 column (1.0 mm×150 mm×1.7 µm) using Acquity UltraPerformance LC system (Waters Co.) coupled to an electrospray (ESI) triple quadrupole mass spectrometer (QTRAP5500; AB SCIEX). The MS/MS analyses were performed in negative ion mode, and the eicosanoids and docosanoids were identified and quantified by multiple reaction monitoring. Calibration curves between 1 and 1000 pg and the LC retention times for each compounds were constructed with synthetic standards. (TIF) [file pone.0089605.s003.tif]
